# Reliability of International Fitness Scale (IFIS) in Chinese Children and Adolescents

**DOI:** 10.3390/children9040531

**Published:** 2022-04-08

**Authors:** Ran Bao, Sitong Chen, Kaja Kastelic, Clemens Drenowatz, Minghui Li, Jialin Zhang, Lei Wang

**Affiliations:** 1 Centre for Active Living and Learning, School of Education, The University of Newcastle, Newcastle 2308, Australia; Ran.Bao@uon.edu.au; 2 Institute for Health and Sport, Victoria University, Melbourne 8001, Australia; sitong.chen@live.vu.edu.au; 3 Andrej Marušič Institute, University of Primorska, Muzejski Trg 2, 6000 Koper, Slovenia; kaja.kastelic@s2p.si; 4 InnoRenew CoE, Livade 6, 6310 Izola, Slovenia; 5 Division of Sport, Physical Activity and Health, University of Education Upper Austria, 4020 Linz, Austria; clemens.drenowatz@ph-ooe.at; 6 Department of Sports Science and Physical Education, The Chinese University of Hong Kong, Hong Kong SAR, China; venuslmh@link.cuhk.edu.hk; 7 School of Physical Education and Sport Science, Fujian Normal University, Fuzhou 350007, China; zhangjialinx@126.com; 8 School of Physical Education and Sport Training, Shanghai University of Sport, Shanghai 200438, China

**Keywords:** the International Fitness Scale (IFIS), reliability, children, adolescents, Chinese

## Abstract

Background and Objectives: It has previously been shown that the International Fitness Scale (IFIS) is a reliable and valid instrument when used in numerous regions and subgroups, but it remains to be determined whether the IFIS is a reliable instrument for use with Chinese children and adolescents. If the reliability of the IFIS can be verified, populational surveillance and monitoring of physical fitness (PF) can easily be conducted. This study aimed to test the reliability of the IFIS when used with Chinese children and adolescents. Methods: The convenience sampling method was used to recruit study participants. In total, 974 school-aged children and adolescents between 11 and 17 years of age were recruited from three cities in Southeast China: Shanghai, Nanjing and Wuxi. The study participants self-reported demographic data, including age (in years) and sex (boy or girl). The participants completed the questionnaire twice within a two-week interval. Results: A response rate of 95.9% resulted in a sample of 934 participants (13.7 ± 1.5 years, 47.4% girls) with valid data. On average, the participants were 13.7 ± 1.5 years of age. The test–retest weighted kappa coefficients for overall fitness, cardiorespiratory fitness, muscle fitness, speed and agility and flexibility were 0.52 (Std. errs. = 0.02), 0.51 (Std. errs. = 0.02), 0.60 (Std. errs. = 0.02), 0.55 (Std. errs. = 0.02) and 0.55 (Std. errs. = 0.02), respectively. Conclusions: The International Fitness Scale was found to have moderate reliability in the assessment of (self-reported) physical fitness in Chinese children and adolescents. In the future, the validity of the IFIS should be urgently tested in Chinese subgroup populations.

## 1. Introduction

Physical fitness (PF) is an important indicator of an individual’s capability to perform physical activity and maintain good health [1]. PF can be defined as the ability of body systems to work together efficiently to allow a human to be healthy and perform daily living activities [2]. Accordingly, PF is a significant marker of health [3,4]. PF is a significant predictor of mortality and morbidity in all-cause [5] and cardiovascular diseases [1,6] and adiposity [7], and the negative impacts of these diseases during childhood and adolescence have negative effects on one’s health in adulthood [1]. In addition, PF was also shown to be related to mental health [8,9], including cognitive functions [10] (e.g., academic performance [11]), depression, anxiety, psychological stress [12] and well-being [7,13]. In addition to health-related PF, which includes cardiorespiratory endurance (CRF), muscular strength and endurance (i.e., muscle fitness, MF), body composition and flexibility (FL), there is also skill-related PF, which includes balance, coordination, speed, power, reaction time and speed and agility [2].

Generally, laboratory and field measurements have been used to evaluate PF [4]. A recent review demonstrated that CRF and MF have been most frequently evaluated in children and adolescents [14]. It was, however, also concluded that standard PF assessments would be needed in the future [14]. Moreover, laboratory or field measurements require time, facilities and equipment, and thus may be less feasible in population-based studies [4,15]. Alternatively, self-reported PF or survey-based methods may be more suitable for the assessment of PF in epidemiological studies. There are several existing fitness scale instruments, such as the Physical Self-Perception Profile (PSPP) [16] or the Self-Reported Fitness (SRFit) scale [17], but the limitation of these scales includes having too many items and targeting specific sub-groups of the population [18].

Therefore, a simple self-administered instrument with no limitations in terms of populations might be suitable for use in population-based surveys. Ortega et al. developed a self-administrated scale to evaluate PF in the general population, which is known as the International Fitness Scale (IFIS) [19]. The IFIS uses a five-point Likert scale (“very good”, “good”, “average”, “poor” and “very poor”) to assess various components of PF [19]. It has been translated into nine languages and consists of five parts, assessing overall PF, cardiorespiratory fitness, muscle fitness, speed and agility as well as flexibility [19]. Previous research demonstrated acceptable construct reliability and validity in European and South American countries in children and adolescents [4,19,20,21,22] as well as adults [18,23,24,25,26]. In comparison with adolescents, the IFIS was found to be more reliable and higher levels of PF were reported in children (3–10 years) [4]. In addition, gender differences in self-reported PF were also observed in the previous study [21]. The differences in age and gender in the reliability and validity of the IFIS suggest that future studies should be directed toward this topic. Although the IFIS has been shown to be a reliable and valid instrument to use to assess self-rated PF in numerous regions and subgroups, it remains to be determined whether the IFIS is a reliable instrument with Chinese children and adolescents. National data from China, however, indicated that only a small percentage (approximately 31.75%) of school-aged children and adolescents rated their PF as “excellent” or “good” [27]. In addition, there has been a decline in PF in young adults over the last several decades [28,29,30]. The decline in PF negatively affects youth health, as discussed above; the monitoring of PF is significant in the design of a strategy to promote a level of PF in Chinese youth. Another justification for this study is that the reliability of the IFIS can be verified, and populational surveillance and the monitoring of PF can easily be conducted. Even though a PF testing system has been built in recent years, a population-based survey is still urgently needed [31]. Considering China’s large population, a Chinese version of the IFIS would be beneficial in the monitoring and promotion of PF. The application of similar methods can also facilitate international comparison of PF. 

Therefore, researchers need a simple and useful instrument to evaluate the levels of PF in various subgroups to monitor and promote the health of Chinese populations. Cultural adaptation, however, requires the reliability of the IFIS to be tested with Chinese children and adolescents. This study, therefore, aimed to determine the reliability of the International Fitness Scale, Chinese-version (IFIS-C), in children and adolescents. 

## 2. Materials and Methods

### 2.1. Participants and Sampling

A pilot study was conducted with children and adolescents, which aimed to evaluate the test–retest reliability and construct the validity of the International Fitness Scale in Chinese children and adolescents. The convenience sampling method was used to select participants, and 1170 school-aged children and adolescents were recruited from Shanghai, Nanjing and Wuxi for this study. An invitation letter was sent to the potential schools, and 7 schools were interested in and agreed to participate in this study. According to previous studies, the sample size in the present study met the standard of reliability [4,20]. In total, 974 school-aged children and adolescents between 11 and 17 years of age provided valid data and were included in the final analysis. This study was approved by the Institutional Review Board (IRB) of the Shanghai University of Sport (Code No.: 102772021RT071). Prior to the questionnaire survey, children and adolescents signed assent forms, and their parents or guardians signed informed consent. 

### 2.2. Measures

#### 2.2.1. Demographics 

Children and adolescents were required to self-report demographic data, including age (year) and gender (boy or girl). Participants were separated by age into children and adolescents using a cut point of 13 years [32]; in this study, adolescents were 13 years old and above [32]. 

#### 2.2.2. Self-Reported Fitness

The International Fitness Scale (IFIS) was used to evaluate the self-estimations of PF using a 5-point Likert scale (“very poor”, “poor”, “average”, “good” and “very good”). Specifically, the IFIS evaluates overall fitness, cardiorespiratory fitness (CRF), muscular strength, speed and agility and flexibility [19]. The English version of the IFIS was separately translated into Chinese (IFIS-C) by two authors who are both proficient in Chinese and English and have research backgrounds in physical activity and fitness promotion. Disagreements on translations between the two authors were resolved by a third author. In addition, back-translation for the IFIS-C into English was conducted by two persons whose source language (English) was their mother tongue [33]. These two persons had no background in PF [33]. 

#### 2.2.3. Data Collection 

The reliability of the International Fitness Scale (IFIS-C) was evaluated through a test–retest design. In order to prevent recall bias, the children and adolescents who participated were required to complete the test–retest within a two-week interval [34]. The procedure used for the reliability test adhered to the COSMIN methodology for systematic reviews of PROMs—user manual (box 6 (reliability)) [35]. On both occasions, the participants completed the measurement under the guidance of the same physical education teacher. The measurement times, timing of the measurement (e.g., before physical education class), measurement place and instructions were similar during both assessment time points. 

#### 2.2.4. Statistical Analysis

The sample size for the test–retest reliability was identified according to previous studies [4,20]. The test–retest reliability of the IFIS-C was calculated for categorical variables using weighted kappa coefficients [36]. Kappa coefficients of less than 0 indicated “no agreement”; kappa = 0.0 to 0.20 indicated “slight agreement”; kappa = 0.21 to 0.40 indicated “fair agreement”; kappa = 0.41 to 0.60 indicated “moderate agreement”; kappa = 0.61 to 0.80 indicated “substantial agreement”; and kappa = 0.81 to 1.00 indicated “almost perfect agreement” [37]. The level of statistical significance was set at *p* < 0.05. SPSS software version 26.0 and Stata MP version 14.1 (Stata Corp LP, College Station, TX, USA) were used to calculate descriptive characteristics and the weighted kappa coefficients in this study. A Confirmatory Factor Analysis (CFA) for the IFIS-C was analyzed using IBM SPSS AMOS 26.0 Graphics. A Confirmatory Factor Analysis (CFA) was conducted to evaluate whether the perceived PF measured using the IFIS-C was consistent with the nature of the construct of the IFIS-C. Factor loadings below 0.3 were not interpreted, and a factor loading of 0.5 or higher was accepted [38]. 

## 3. Results

After deleting questionnaires with missing data, a response rate of 95.9% resulted in a sample of 934 children and adolescents (47.4% girls) with valid data that were included in the analyses. The sample consisted of 390 children (41.8% girls) and 544 adolescents (58.2% girls). The average age was 13.7 ± 1.5 years. 

**Figure 1:**
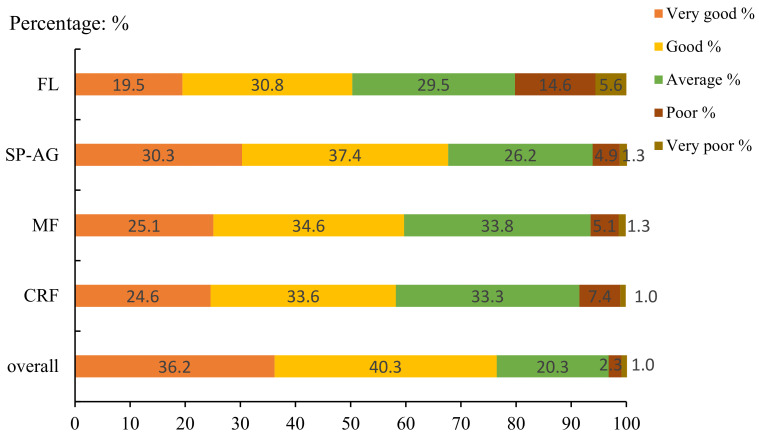
Distribution of responses by categories of self-reported physical fitness in children (CRF = cardiorespiratory fitness; MF = muscular fitness; SP–AG = speed and agility; FL = flexibility).

**Figure 2:**
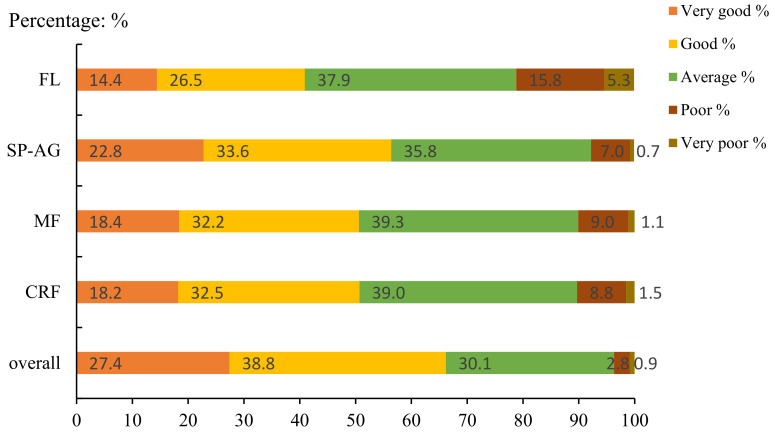
Distribution of responses by categories of self-reported PF in adolescents (CRF = cardiorespiratory fitness; MF = muscular fitness; SP–AG = speed and agility; FL = flexibility).

Figure 1 and Figure 2 show the results of self-rated fitness. Overall, most children rated their fitness as “good”, and most adolescents rated their fitness as “average”. Meanwhile, no adolescents reported their SP–AG as “very poor”. 

Table 1 shows the overall weighted kappa values of the test–retest reliability of the self-reported IFIS-C. Test–retest weighted kappa coefficients for overall, CRF, MF, SP–AG and FL were 0.52 (Std. errs. = 0.02), 0.51 (Std. errs. = 0.02), 0.60 (Std. errs. = 0.02), 0.55 (Std. errs. = 0.02) and 0.55 (Std. errs. = 0.02), respectively, which indicate the moderate reliability of the IFIS-C [36]. The highest weighted kappa value of IFIS-C was found for the MF score (weighted kappa = 0.60). 

Table 2 shows the weighted kappa of the IFIS-C by gender. The overall weighted kappa of the IFIS-C showed moderate reliability, with a somewhat better weighted kappa value observed in girls (0.54) than in boys (0.48). Specifically, the highest weighted kappa value observed in boys was 0.58 (Std. errs. = 0.03) for MF, and the lowest weighted kappa value was 0.48 (Std. errs. = 0.03) for overall fitness. In girls, the highest weighted kappa value was 0.60 (Std. errs. = 0.03) for FL and MF, and the lowest weighted kappa value was 0.49 (Std. errs. = 0.03) for CRF.

The weighted kappa of the IFIS-C in children and adolescents are shown in Table 3. Moderate reliability was indicated for all of the components of the IFIS-C in the group of children and adolescents. Nevertheless, higher weighted kappa values were observed in children compared to adolescents. In children, the highest coefficient of weighted kappa was 0.69 (Std. errs. = 0.04) for MF, and the lowest coefficient of weighted kappa was 0.61 for overall fitness and CRF. In adolescents, the highest coefficient of weighted kappa was 0.52 (Std. errs. = 0.03) for MF, and the lowest coefficient of weighted kappa was 0.42 (Std. errs. = 0.03) for overall fitness and CRF. In addition, the internal consistency was accepted, and the alpha coefficient was 0.719 (data not shown in the tables). 

The goodness-of-fit for the IFIS-C is outlined in Table 4. The fit indices were 0.95 or higher, and the RMSEA and SRMR were below 0.08, which indicate a good model fit [39]. Indices of model fit indicated that the IFIS-C showed a good model fit in Chinese children and adolescents. However, the RMSEA was 0.114 in girls, which was not acceptable according to the cutoff of below 0.08. Table 5 shows the factor loadings, CR and AVE of the IFIS-C. Factor loadings of CRF, MF and SP–AG were all above 0.5, which indicated acceptable values. However, the factor loading of flexibility was below 0.5. Regarding CR and AVE, CR was accepted by gender and age subgroups. However, values of AVE were only accepted in boys and adolescents. 

## 4. Discussion

To the authors’ knowledge, this study was the first to evaluate the reliability of the IFIS in China. Overall, the results indicate the moderate reliability of the IFIS-C in Chinese children and adolescents with weighted kappa values for different sub-measures of the IFIS-C ranging from 0.51 to 0.60. In terms of subgroups, the weighted kappa values were slightly higher in girls and children than in boys and adolescents, respectively. In addition, there was a lower reliability for overall fitness in comparison to other components of the IFIS-C, and MF showed a greater reliability in Chinese children and adolescents. 

Several previous studies suggested that the IFIS has moderate reliability in children and adolescents [4,19,20,21,22]. The overall weighted kappa coefficients in this study are comparable to previously reported weighted kappa coefficients between 0.54 and 0.65 in European children and adolescents [19]. Furthermore, the weighted kappa coefficient for MF was similar to that found in Francisco’s study [19]. Another study, however, reported a range of 0.52–0.67 in adolescents, which was higher than in this study (the weighted kappa of this study ranged from 0.45 to 0.56) [22]. Higher weighted kappa coefficients in children and adolescents were also reported in two other studies that used the Spanish version of the IFIS, which were 0.775 to 0.847 [20] and 0.64 to 0.80 [21], respectively. These differences may be attributed to the variability in physical activity and fitness level across study populations, as previous studies showed that well-designed physical activity can improve the perceived PF in adolescents [40]. The results of the Confirmatory Factor Analysis (CFA) indicated that the model fit was not acceptable in girls, which revealed that the IFIS-C had poor construct validity in Chinese girls. However, the reason for this might be the small sample size of girls in this study. The lower level of physical fitness in Chinese school-aged girls in comparison with that in boys may also have contributed to these results [27]. Therefore, future studies with larger sample sizes are needed to further examine the validity of the IFIS-C.

This study also showed that few children and adolescents consider their PF as “poor” or “very poor”, which is consistent with previous research [21]. Sex-specific analyses also showed higher self-estimations of PF in boys than in girls, except in terms of flexibility, which was consistent with previous studies [19,21]. Potential contributors to these observed differences may be maturity status [41], morphological characteristics (different somatotypes) [42] and physiological traits [21,43]. Differences in the types of physical activity performed between boys and girls may also contribute to differences in perceived PF [22,44], as different types of physical activities can enhance various aspects of PF. For example, boys prefer ball sports that can increase strength and SP–AG fitness, while girls are more willing to participate in dance or gymnastics that can increase flexibility [44]. Despite these differences, the IFIS-C can be considered a reliable instrument for use in determining PF by sex in Chinese children and adolescents.

With regard to age, adolescents reported lower self-estimations of fitness than children in this study, which has been shown previously [4]. A decline in PF across different grades has also been reported in a large-scale study of Chinese children and adolescents [27]. Compared with other components of the IFIS-C, Chinese children and adolescents reported higher self-estimated overall fitness, which was similar to the results of previous studies in Brazilian, Spanish and Colombian adolescents [4,20,21]. Notably, existing evidence revealed that PF was closely associated with motor competence [45]. Considering that daily physical activity generally involves components of strength, speed or flexibility [4], the perception of children’s and adolescents’ motor performance is closely related to all of the components of PF acquired in daily physical activities [4]. Therefore, participants reported higher self-estimated overall fitness in comparison with other components of the IFIS-C. 

In general, the IFIS is a reliable instrument that can be used to evaluate the overall level of PF in population-based studies (i.e., epidemiological studies), and there is a need to test the reliability and validity of the IFIS in other age subgroups (i.e., youth, young adults and old adults) in different regions of China. Although this reliability study was conducted with Chinese children and adolescents and had a large sample size, several limitations should be taken into consideration. Firstly, due to various circumstances (i.e., COVID-19 restrictions and the fact that measurements require time, facilities and equipment), it was not possible to conduct a field-based PF evaluation. Additional research, therefore, is necessary to determine the validity of the IFIS-C in Chinese children and adolescents. Secondly, validity and reliability need to be determined in other age groups to promote a national use in China. Thirdly, the sample was taken from eastern China, and reliability and validity studies should be conducted in different regions in China due to differences in PF levels in these regions. 

## 5. Conclusions 

Overall, this study showed that the IFIS-C is a reliable instrument for the assessment of PF in Chinese children and adolescents. The lower reliability of overall fitness also emphasizes the need to separately assess various subcomponents of PF in Chinese children and adolescents to gain accurate insight into the key components of PF.

## Figures and Tables

**Table 1:** Reliability of the International Fitness Scale.

	Test One, Mean (SD)	Test Two, Mean (SD)	Weighted Kappa	Std. Errs.
Overall	3.97 ± 0.88	3.95 ± 0.92	0.52	0.02
CRF	3.64 ± 0.94	3.71 ± 0.93	0.51	0.02
MF	3.66 ± 0.93	3.67 ± 0.95	0.60	0.02
SP–AG	3.79 ± 0.93	3.84 ± 0.91	0.55	0.02
FL	3.35 ± 1.10	3.40 ± 1.09	0.55	0.02

SD = standard deviation; CRF = cardiorespiratory fitness; MF = muscular fitness; SP–AG = speed and agility; FL = flexibility.

**Table 2:** Weighted kappa of International Fitness Scale by gender.

		Test One, Mean (SD)	Test Two, Mean (SD)	Weighted Kappa	Std. Errs.
Boys	overall	4.11 ± 0.82	4.07 ± 0.91	0.48	0.03
	CRF	3.76 ± 0.96	3.84 ± 0.95	0.51	0.03
	MF	3.78 ± 0.96	3.81 ± 0.99	0.58	0.03
	SP–AG	3.95 ± 0.96	3.96 ± 0.95	0.55	0.03
	FL	3.23 ± 1.10	3.26 ± 1.12	0.51	0.03
Girls	overall	3.82 ± 0.90	3.81 ± 0.93	0.54	0.03
	CRF	3.51 ± 0.91	3.56 ± 0.89	0.49	0.03
	MF	3.52 ± 0.88	3.51 ± 0.87	0.60	0.03
	SP–AG	3.62 ± 0.86	3.70 ± 0.84	0.53	0.03
	FL	3.47 ± 1.08	3.57 ± 1.04	0.60	0.03

SD = standard deviation; CRF = cardiorespiratory fitness; MF = muscular fitness; SP–AG = speed and agility; FL = flexibility.

**Table 3:** Weighted kappa of International Fitness Scale in children and adolescents.

		Test One, Mean (SD)	Test Two, Mean (SD)	Weighted Kappa	Std. Errs.
Children (n = 390)	overall	4.08 ± 0.86	4.11 ± 0.90	0.61	0.04
	CRF	3.73 ± 0.95	3.84 ± 0.93	0.61	0.03
	MF	3.77 ± 0.93	3.81 ± 0.93	0.69	0.04
	SP–AG	3.91 ± 0.93	3.98 ± 0.90	0.64	0.04
	FL	3.44 ± 1.13	3.48 ± 1.12	0.66	0.03
Adolescents (n = 544)	overall	3.89 ± 0.87	3.83 ± 0.92	0.44	0.03
	CRF	3.57 ± 0.94	3.62 ± 0.93	0.42	0.03
	MF	3.58 ± 0.93	3.57 ± 0.95	0.52	0.03
	SP–AG	3.71 ± 0.92	3.74 ± 0.90	0.48	0.03
	FL	3.29 ± 1.06	3.34 ± 1.07	0.47	0.03

SD = standard deviation; CRF = cardiorespiratory fitness; MF = muscular fitness; SP–AG = speed and agility; FL = flexibility.

**Table 4:** Goodness-of-fit indices for the IFIS-C in Chinese children and adolescents.

Statistics	Boys	Girls	Children	Adolescents	Overall
χ^2^	1.157	13.395	4.067	1.018	3.525
df	2	2	2	2	2
p	0.561	0.001	0.131	0.601	0.172
χ^2^/df	0.579	6.697	2.033	0.509	1.763
SRMR (95% CI)	0.011	0.043	0.027	0.010	0.015
RMSEA (95% CI)	0.000	0.114	0.052	0.000	0.029
GFI	0.999	0.985	0.995	0.999	0.998
CFI	1.000	0.971	0.994	1.000	0.999
NFI	0.998	0.966	0.988	0.998	0.997
TLI	1.004	0.912	0.982	1.004	0.996
IFI	1.001	0.971	0.994	1.001	0.999

Abbreviation: df = degree of freedom; SRMR = standardized root means square residual; RMSEA = root mean square error of approximation; CI = confidence interval; GFI = goodness-of-fit index; CFI = comparative fit index; NFI = normed fit index; TLI = Tucker–Lewis index; IFI = incremental fit index.

**Table 5:** Factor loadings, CR and AVE of the IFIS-C in Chinese children and adolescents.

Statistics	Boys	Girls	Children	Adolescents	Overall
CRF	0.78	0.69	0.76	0.73	0.74
MF	0.80	0.68	0.68	0.80	0.75
SP–AG	0.77	0.74	0.74	0.78	0.77
FL	0.47	0.47	0.34	0.49	0.43
CR	0.80	0.74	0.73	0.80	0.77
AVE	0.52	0.43	0.43	0.51	0.47

Abbreviation: CRF = cardiorespiratory fitness; MF = muscle fitness; SP–AG = speed and agility; FL = flexibility; CR = composite reliability (>0.7); AVE = average variance extracted (>0.5) [37].

## Data Availability

Not applicable.
